# *Notes from the Field*: Xylazine, a Veterinary Tranquilizer, Identified as an Emerging Novel Substance in Drug Overdose Deaths — Connecticut, 2019–2020

**DOI:** 10.15585/mmwr.mm7037a5

**Published:** 2021-09-17

**Authors:** Shobha Thangada, Heather A. Clinton, Sarah Ali, Jacqueline Nunez, James R. Gill, Robert F. Lawlor, Susan B. Logan

**Affiliations:** ^1^Injury and Violence Surveillance Unit, Connecticut Department of Public Health, Hartford, Connecticut; ^2^Injury Prevention Center, Connecticut Children’s, Hartford, Connecticut; ^3^New England High Intensity Drug Trafficking Area; ^4^Connecticut Office of the Chief Medical Examiner, Farmington, Connecticut

Xylazine, a clonidine analog, is a nonopioid veterinary tranquilizer not intended for human use. Recreational drugs such as cocaine, heroin, and fentanyl are often adulterated with agents such as xylazine to enhance drug effects or increase street value by increasing net weight (*1*). Xylazine is known to cause hypotension and bradycardia when used in humans (*2*). Although not a controlled substance in the United States, xylazine cannot be purchased without a veterinary license. Misuse of xylazine was reported in Puerto Rico in the early 2000s (*3*). Recreational use of xylazine can occur via oral ingestion, inhalation or sniffing, or intravenous injection; however, injection is the most common route of administration (*2*). The effects of xylazine when used contemporaneously with other illicit drugs such as heroin, cocaine, and fentanyl are still not widely known (*2*). No antidote is recommended for the effects of xylazine overdose (*4*). One recent study suggests that high doses of naloxone might reverse the effects of a clonidine overdose (*5*). However, given that this finding is from a single study of a small cohort of pediatric patients, it might not be generalizable to the broader population (*5*). Furthermore, no reports specific to xylazine and naloxone exist regarding reversal of effects.

Routine drug screening is conducted for all suspected drug overdose deaths investigated by the Connecticut Office of the Chief Medical Examiner, and xylazine has been included in toxicology panels since 2013. Antemortem and postmortem specimens are collected by the Office of the Chief Medical Examiner; toxicology analysis is performed using liquid chromatography time-of-flight mass spectrometry and liquid chromatography-tandem mass spectrometry by National Medical Services Laboratories in Horsham, Pennsylvania.

During 2019, a total of 1,200 deaths from unintentional drug overdoses were reported in Connecticut; test results for 70 (5.8%) decedents were positive for xylazine. During January–July 2020, 666 deaths from drug overdoses were reported in Connecticut; test results for 76 (11.4%) were positive for xylazine. Among 146 xylazine-positive deaths during 2019 and 2020 (Table), test results for all but one (99.3%) were positive for fentanyl. Xylazine-associated deaths occurred primarily among males (80.9%) and non-Hispanic White persons (74.0%). Mortality was highest among persons aged 25–34 years (28.1%), followed by those aged 35–44 (26.7%) and ≥55 years (23.3%). Fifty-seven percent of xylazine-associated deaths occurred at home, which was also the predominant location of overdose (75.3%). Twenty-six percent of deaths occurred at the hospital; naloxone was administered 18.5% of the time. Sixty-seven percent of decedents had a prior history of substance misuse. Based on drug paraphernalia found at the location of overdose, routes of administration were injection (39.7%), unknown (29.5%), snorting (15.1%), smoking (13.7%), and ingestion (4.1%). Toxicology analysis revealed that 85.6% of xylazine-fentanyl deaths included other substances: cocaine (34.2%), heroin (30.1%), benzodiazepines (26.0%), ethanol (22.6%), and gabapentin (12.3%).

**TABLE:** Characteristics, circumstances, and co-occurring substances among overdose decedents with xylazine detected in postmortem toxicology — Connecticut, 2019–July 2020

Characteristic/Circumstance	No. (%)
**Total**	146 (100)
**Sex**	
Male	118 (80.9)
Female	28 (19.2)
**Race/Ethnicity**	
White, non-Hispanic	108 (74.0)
Black, non-Hispanic	10 (6.8)
Hispanic*	25 (17.1)
Other, non-Hispanic^†^	2 (1.4)
Unknown	1 (0.7)
**Age group, yrs**	
<25	8 (5.5)
25–34	41 (28.1)
35–44	39 (26.7)
45–54	24 (16.4)
≥55	34 (23.3)
**Location of injury**	
Home	110 (75.3)
Motel/Hotel	11 (7.5)
Residential institutes	6 (4.1)
Motor vehicle	4 (2.7)
Other^§^	15 (10.3)
**Location of death**	
Home	83 (56.8)
Hospital (DOA/ED/Inpatient)	38 (26.0)
Motel/Hotel	10 (6.8)
Friend's house	8 (5.5)
Other^¶^	7 (4.8)
**History of substance misuse reported (opioid/nonopioid)**	
Evidence	98 (67.1)
No evidence	48 (32.9)
**Naloxone administration**	
Naloxone given	27 (18.5)
Naloxone not given or unknown	119 (81.5)
Route of administration**	
Injection	58 (39.7)
Snorting	22 (15.1)
Smoking	20 (13.7)
Ingestion	6 (4.1)
Unknown	43 (29.5)
**Co-occurrence of fentanyl**	145 (99.3)
Fentanyl only	21 (14.4)
Fentanyl plus one or more other substances	124 (85.6)
Fentanyl and cocaine^††^	50 (34.2)
Fentanyl and heroin^††^	44 (30.1)
Fentanyl and alcohol^††^	33 (22.6)
Fentanyl and benzodiazepines^††^	38 (26.0)
Fentanyl and gabapentin^††^	18 (12.3)

**Source**: Office of the Chief Medical Examiner, Farmington, Connecticut.

**Abbreviations**: DOA = dead on arrival; ED = emergency department.

* Hispanic or Latino.

^†^ Non-Hispanic American Indian or Alaska Native, Asian, or Native Hawaiian or Other Pacific Islander.

^§^ Park; vacant places; outdoor area; or unknown.

^¶^ Motor vehicle; park; halfway house; or outdoor area.

** Based on drug paraphernalia present at the scene.

^††^ Subgroups total to more than 100% because test results for some decedents were positive for multiple substances.

These findings demonstrate a rising prevalence of xylazine-involved unintentional overdose deaths in Connecticut. The combination of xylazine with opioids or other recreational drugs might increase their toxic effects by potentiating sedation and causing respiratory depression, hypotension, and bradycardia (*1*,*2*). Awareness among health care professionals of issues related to xylazine is important because xylazine intoxication is unaffected by standard doses of naloxone, which is the usual treatment for suspected opioid intoxication (*6*). Xylazine intoxication might require additional interventions and appropriate supportive measures, which include blood pressure support with intravenous fluids, atropine, and extended hospital observation because of cardiac effects (*7*). The effects of xylazine when combined with fentanyl, heroin, or cocaine need further research to clarify adverse interactions and identify effective therapies. Given that xylazine is often reported in combination with fentanyl and heroin, naloxone administration is still advisable for suspected intoxications involving xylazine to treat the effects of opioids.

With funding from CDC, the State Public Health Laboratory is now capable of testing for xylazine and other illicit drugs in urine samples persons who experience nonfatal drug overdoses; the goals are to improve the timeliness of xylazine detection and better enable tracking of emerging drug trends in Connecticut. The testing of seized drugs by the Division of Scientific Services’ forensic laboratory allows law enforcement agencies to track specific distributors of fentanyl-xylazine combinations. Because recent xylazine overdoses have occurred in Rhode Island and New Jersey, communicating and collaborating with other states will help identify drug trends, provide information that will enhance surveillance efforts to track emerging substances and guide prevention initiatives, and aid health care professionals to treat patients in a more timely and effective manner.
